# From *warrior genes* to translational solutions: novel insights into monoamine oxidases (MAOs) and aggression

**DOI:** 10.1038/s41398-021-01257-2

**Published:** 2021-02-18

**Authors:** Alexios-Fotios A. Mentis, Efthimios Dardiotis, Eleni Katsouni, George P. Chrousos

**Affiliations:** 1 Public Health Laboratories, Hellenic Pasteur Institute, Vas. Sofias Avenue 127, 115 21 Athens, Greece; 2 Department of Neurology, University of Thessaly, Panepistimiou 3, Viopolis, 41 500 Larissa, Greece; 3 Department of Experimental Psychology, Oxford University, Oxford, UK; 4 University Research Institute of Maternal and Child Health and Precision Medicine, National and Kapodistrian University of Athens, Medical School, Aghia Sophia Children’s Hospital, Livadias 8, 115 27 Athens, Greece; 5 UNESCO Chair on Adolescent Health Care, Athens, Greece

**Keywords:** Molecular neuroscience, Psychiatric disorders, Clinical pharmacology

## Abstract

The pervasive and frequently devastating nature of aggressive behavior calls for a collective effort to understand its psychosocial and neurobiological underpinnings. Regarding the latter, diverse brain areas, neural networks, neurotransmitters, hormones, and candidate genes have been associated with antisocial and aggressive behavior in humans and animals. This review focuses on the role of monoamine oxidases (MAOs) and the genes coding for them, in the modulation of aggression. During the past 20 years, a substantial number of studies using both pharmacological and genetic approaches have linked the MAO system with aggressive and impulsive behaviors in healthy and clinical populations, including the recent discovery of *MAALIN*, a long noncoding RNA (lncRNA) regulating the *MAO-A* gene in the human brain. Here, we first provide an overview of the MAOs and their physiological functions, we then summarize recent key findings linking MAO-related enzymatic and gene activity and aggressive behavior, and, finally, we offer novel insights into the mechanisms underlying this association. Using the existing experimental evidence as a foundation, we discuss the translational implications of these findings in clinical practice and highlight what we believe are outstanding conceptual and methodological questions in the field. Ultimately, we propose that unraveling the specific role of MAO in aggression requires an integrated approach, where this question is pursued by combining psychological, radiological, and genetic/genomic assessments. The translational benefits of such an approach include the discovery of novel biomarkers of aggression and targeting the MAO system to modulate pathological aggression in clinical populations.

## Introduction

Aggression is an evolutionarily conserved, complex set of behaviors aimed at inflicting physical and/or emotional harm to others. Though adaptive in certain situations, aggressive behaviors or traits that deviate from normative standards (i.e., labeled as pathological^1^) can lead to detrimental personal and societal consequences, as is the case for antisocial behavior. Far from being a unitary behavioral construct, aggression is often hard to operationalize and measure in the laboratory setting^2,3^. Part of this complexity is also explained by the fact that the conceptualization of aggression has changed throughout history and across cultures^4^.

Despite the inherent complex nature of aggression, numerous efforts have been made to elucidate the neurobiological and genetic underpinnings of aggressive behavior across the animal kingdom (for a review, see refs. ^5–9^). On this front, diverse brain areas, neural networks, neurotransmitters, hormones, and candidate genes have been experimentally related to aggressive and antisocial behavior in humans and other animals. Among these, monoamine oxidases (MAOs) have received substantial attention over the past 20 years. The renewed interest in aggression and MAOs can be traced back to 1993, when Brunner et al. reported abnormal behavioral manifestations, including overt aggressive and violent behavior, in a Dutch family with an X-linked nonsense mutation in the *MAO-A* gene^10^ (Brunner syndrome). As we discuss below, similar behavioral phenotypes have been observed in mice with *MAO-A* gene mutations that mimic that of the human Brunner syndrome. In 2013, 20 years later, Piton et al. reported the second case of *MAO-A* mutations in a small family with a behavioral phenotype similar to the one originally described by Brunner^11^, and this was followed by the third report of two Australian families^12^.

Because of the chief role of MAOs in the metabolism of key neurotransmitters involved in aggressive behavior, most notably serotonin (5-hydroxytryptamine; 5-HT)^13,14^, it is not surprising that a substantial body of research has linked aggressive phenotypes with the MAO system. Thus, human genetic studies associated aggressive traits with specific allelic variations in the genes that encode the MAOs^6,15–18^. In addition, humans and mice with an absent or dysfunctional gene that codes for the isoform A of the enzyme exhibited increased 5-HT levels and manifested aggressive and impulsive behaviors^15,19^. Indeed, these results inspired both the scientific community and media outlets to refer to *MAO-A* as the *warrior* or *criminal* gene^20^. On the other hand, pharmacological studies characterized the behavioral changes associated with the administration of selective and nonselective MAO inhibitors (MAOIs), which, interestingly, are widely prescribed for the treatment of a variety of mental disorders, including anxiety, depression, posttraumatic stress disorder (PTSD), and Parkinson disease^21^. Although initial pharmacological studies supported a role for MAOIs in the reduction of aggressive phenotypes, data from these studies were hard to interpret because of the side effects of MAOIs, and because of their impact on a myriad of unrelated behaviors^21^.

Here, we first provide a general overview of MAOs and their physiological functions. Then, we summarize recent key findings linking MAO-related enzymatic and gene expression activity and aggressive behavior, and we provide novel insights into the mechanisms underlying this association. Using the existing experimental evidence as a foundation, we discuss the translational implications of these findings in clinical practice and also highlight what we consider outstanding conceptual and methodological questions in the field.

## Overview of the MAO system

In humans and rodents, the MAO-A and MAO-B isoenzymes are encoded by the homonymous genes *MAO-A* and *MAO-B*, respectively. The validity of neurobehavioral studies linking MAO and aggression has been questioned in animal models, such as the zebrafish, which only carry one *Mao* gene^22^. *MAO-A* and *MAO-B* are both on the X chromosome and present similar intron–exon organization; however, they show discrepancies in their core promoter regions^23–25^. These differences in the regulation of *MAO* genes may explain their distinct responses to certain hormones and levels of gene expression across brain areas^26^ and the extent to which MAO-A and MAO-B modulate antisocial and aggressive behaviors (discussed below).

In the central nervous system, MAOs break down monoamine neurotransmitters, including the catecholamines dopamine, adrenaline, and noradrenaline, histamine, and serotonin (5-HT)^25–27^. Thus, MAO inhibitors (MAOIs) have been commonly prescribed to treat disorders or conditions characterized by reduced levels of monoamine neurotransmitters, including affective disorders (e.g., anxiety and depression), Alzheimer’s disease (AD), and Parkinson’s disease^26^. The MAO-A and MAO-B isoenzymes differ in key anatomical, biochemical, and functional aspects (for a comprehensive review, see ref. ^26^). Within the nervous system, there are region- and cell-dependent differences in MAO activity. In humans, increased MAO activity has been observed in the basal ganglia and hypothalamus and reduced activity in the cerebellum and neocortex^28^. Regarding cell-specific activity, MAO-A is mostly expressed in catecholaminergic neurons, while MAO-B shows a higher presence in serotonergic neurons and astrocytes^29^. From a functional standpoint, both MAO-A and MAO-B are active toward dopamine, adrenaline, and noradrenaline^26^. However, whereas MAO-A seems to bear a larger share of the responsibility in the metabolism of 5-HT, MAO-B mostly catalyzes the oxidation of benzylamine and 2-phenylethylamine^26^.

Finally, because we discuss studies in both humans and rodents, it is worth mentioning that the MAO system presents some differences across species^26^. For instance, while both MAO-A and MAO-B catalyze dopamine, adrenaline, and noradrenaline oxidation in humans, in rats MAO-A participates primarily in dopamine metabolism^30^.

## The complexity of aggressive behavior

The discovery by Brunner et al. that mutations in the human MAO-A gene lead to aggressive phenotypes^10^ and that MAOs’ primary function is in the metabolism of 5-HT and other monoamine neurotransmitters, justify a strong scientific interest in the modulatory role of the MAO system in adaptive and pathological aggression^13,14^. Before we discuss key findings supporting this role, we would like to underscore that aggression is a complex, multifaceted behavioral construct supported by a similarly complex neurobiological system^31^. This complexity raises a few considerations when interpreting the results discussed here. First, the MAO system is one of many physiological systems that regulate the behavioral manifestations of aggression and antisocial behavior. Second, this system interacts with a myriad of factors that underlie aggression, including political, socioeconomic, cultural, medical, and psychological factors^32^. Third, caution should be made when extrapolating results from animal models to humans, given the core differences in the monoamine system across species^26^. Finally, because aggression can be defined and operationalized in multiple ways^2^, one should always bear in mind the conceptual framework used in any given study (Box 1).

Box 1 Theoretical aspects of aggression As for most behaviors, the underpinnings of aggression are complex and include neurochemical, hormonal, genetic, social, and psychological factors. From a conceptual and operational perspective, aggression in humans and animal models has been defined and measured in different ways throughout history. Although aggressive behavior is deliberate by definition, the motivating factors, modalities, and the social or biological context in which this behavior takes place to trigger a variety of forms of aggression. In the laboratory setting, a proper theoretical conceptualization of aggression is necessary for the extrapolation of results and impact on the clinical practice.

In animals, multiple frameworks have been proposed to classify the different types of aggression, including defensive aggression, offensive aggressive behavior, and indiscriminate or irritable aggression (i.e., in response to a nonspecific provocation)^101^. In addition, depending on the intended goal or context, where the aggression takes place, authors like Moyer have proposed the existence of predatory, inter-male, fear-induced, sex-related, maternal, and instrumental aggression^102^. These different versions of aggressive behavior are also thought to be supported by differentiated biological mechanisms. Aggression in the animal model is assessed via behavioral tasks or situational tests that vary depending on the species and type of aggression measured^103^.

In humans, aggressive behavior may be more nuanced and complex than in other animal species. Theories of human aggression and taxonomies, such as the one proposed by Krahe^104^, refer to different subtypes of aggression depending on the response modality (e.g., verbal, physical), immediacy (direct or indirect), visibility (overt or covert), instigation (unprovoked or retaliative), type of harm (physical or psychological), and so on. Classic models also include a distinction between instrumental and hostile aggression^105^. Whereas instrumental aggression aims to achieve the desired consequence that goes beyond the aggressive act (e.g., non-aggressive incentives like money, power, or advantage, or destroying someone’s social status and relationships (so-called relational aggression), hostile aggression occurs when the goal is solely to cause harm in the victim. From an evolutionary perspective, others have classified aggression into proactive (i.e., a planned attack associated with an internal or external reward) and reactive (i.e., an aggressive response to a threatening event aimed to eliminate the aversive or provoking stimulus)^106^. In social contexts, we can also consider direct aggression (occurring in direct interactions between the aggressor and the victim) and indirect aggression (without direct contact, caused via a third party or object)^107^. Examples of the latter would be to harm someone’s reputation or status. Historically, the assessment of the genetic and environmental contributions to aggression has been conducted in cross-sectional or longitudinal studies using twin registries or case controls. In this context, aggression is typically measured via questionnaires, aggression scales, or objective data from public registries^108^.

From a clinical perspective, and according to the American Psychological Association (APA), aggressive behavior can be considered pathological when it is either part of a longstanding repertoire of destructive behaviors or consists of a sudden, exaggerated reaction to a real or perceived provocation^109^. In some cases, this pattern of behavior may be the manifestation of a psychiatric disorder, including psychosis, PTSD, antisocial personality disorder, or a consequence of substance use (e.g., alcohol)^109^.

## The link between MAOs and aggression: where are we?

In this section, we briefly summarize the main findings linking the MAOs to aggressive/antisocial behavior in humans and other species. Using classic published data and recent findings as a starting point, we next identify some of the outstanding questions in the field, suggest novel research avenues, and identify conceptual and technical obstacles to overcome in the future.

### Experimental evidence in humans and animal models

#### Pharmacological approach

Pharmacological studies linking MAOs and aggression have traditionally focused on the behavioral effects of MAOIs, which are widely prescribed for the treatment of a variety of neurocognitive disorders, including depression, posttraumatic stress disorder, or Parkinson disease^21^. Also, nonselective MAOIs or selective MAO-B inhibitors have been used to manage suicidal tendencies and impulsive–aggressive behavior^21,33^, whereas selective MAO-A inhibitors have been employed in Antisocial and Borderline Personality Disorders and yielded conflicting results^34^. Further research in human subjects is required to determine the global effect of MAOIs. For instance, selegiline, a selective, irreversible inhibitor of MAO-B commonly used to treat symptoms in patients with Parkinson’s disease, has been shown to influence MAO-A activity in a dose-dependent manner^35^.

In mice, prenatal suppression of MAO-A leads to increased aggression in adulthood after acute administration of MAO-A inhibitors, which suggests a potential prenatal sensitization mechanism^36^. In adolescent (but not neonatal) mice, selective MAO-A inhibition also leads to increased aggression^37^. Chronic suppression of MAO-A, on the other hand, decreased aggression in adult mice^38^. Similarly, in adult mice and rats, nonselective inhibitors of MAO-A and MAO-B suppressed rather than facilitated aggression^21,39,40^. These results indicate that MAO inhibitors may exert different influences on aggression, depending on developmental stage, selectivity, and dosing regimen.

Although initial evidence from pharmacological and pharmacogenetic studies supported a role for MAOIs in the modulation of aggressive phenotypes, data from these studies were often misinterpreted^41^ and were hard to extrapolate because of the side effects of such manipulations and their impact on physiological responses and a myriad of unrelated behaviors and, including responses to stress^21^.

#### Genetic studies

Substantive evidence supporting the role of the *MAO* genes in aggression comes from studies in knockout (KO) mice. Selective knockout models for the *MAO-A* gene, for instance, exhibited increased aggressiveness compared to their wild-type counterparts^42^. Specifically, adult KO mice housed in groups exhibited signs of aggressive behavior, including bite wounds and faster attack to intruders in resident-intruder tests, as compared with control mice^42^. These effects were likely mediated by reduced functionality of the serotoninergic system in sensitive developmental periods of *MAO-A*-deficient mice^14,26,42,43^. To this end, pharmacological MAO-A blockade or 5-HT inhibition during the early postnatal period in mice—from postnatal-day (P-) 21—but not during peri-adolescence (P22–P41)—led to a similar behavioral phenotype in adulthood, characterized also by anxiety and depressive behaviors^37^. Conversely, MAO-A blockade in the peri-adolescence, but not early postnatal period, led to increased aggression in adulthood^37^. Of note, MAO-A “hypomorphic” mice, i.e., mice with partial but not a total loss of MAO-A, did not exhibit increased overt aggression but had reduced social interactions and perseverative responses^44^. In contrast to complete *MAO-A* KO mice, *MAO-B* KO mice did not show decreased serotoninergic function or overt aggressive behavior^25,43^. However, results from genetic or pharmacological manipulations of *MAO* and MAO, respectively, should be interpreted in light of the broader cognitive and emotional impairments associated with deficiencies in the MAO system^13^.

As with mice, *MAO-A* deficiency in humans was associated with an aggressive behavioral phenotype. Brunner et al. reported mental retardation and abnormal behaviors, including impulsive aggression and antisocial behaviors in men of a Dutch family affected by a nonsense mutation in the eighth exon of the *MAO-A* gene^10^. Although all cases of Brunner syndrome were members of the same family, jeopardizing further generalization of the findings^45^, this report sparked a lot of attention and research on the link between MAO and aggressive behavior. Further clinical evidence in humans was reported in studies focusing on the polymorphic variants of *MAO-A*, specifically variable-number tandem repeat (VNTR) polymorphisms or adjacent repetitions of nucleotide sequences. The number of tandem repeats (ranging 2–5 repeats) in the *MAO-A* gene has functional implications, given that the 2- and 3-repeat alleles (i.e., low-expressing alleles) of the gene, lead to lower enzyme activity than the 4-repeat variant^13,46^. Consequently, several studies identified a link between the 2- and 3-repeat alleles and aggressive traits, psychopathy, and criminal behavior^13,15^. In addition, several studies demonstrated that maltreated and abused children with the low *MAO-A* expression allele exhibited behavioral problems in adulthood, while high expression seemed to be protective from such behavioral phenotype in this population^47–49^. The role of *MAO-A* expression in aggressive behavior should be interpreted in light of the multifactorial nature of aggression, where other genetic and epigenetic factors simultaneously influence this behavioral phenotype^49^. For instance, in female adolescent populations, where X inactivation and other epigenetic transformations may have a profound impact, the social risk appeared to be a prerequisite for later associations between *MAO-A* (4-repeat allele) and aggression^50^. It has also been suggested that *MAO-A* and testosterone interactions on aggression are related to modified gene expression^51^.

Genetic studies in humans provide evidence that *MAO-A* is linked to aggression but only when other environmental factors are also present during development (e.g., abuse, stressors). Other lines of research have demonstrated that the gene product of *MAO-A* (rather than the gene per se) influences violent traits. For instance, cortical and subcortical MAO-A activity in vivo -measured with positron emission tomography (PET)- was negatively associated with trait aggression^52^. Likewise, a [^11^C]harmine PET study revealed that increased MAO-A binding in the PFC, indicating higher levels of MAO-A in this brain area, was negatively associated with maladaptive personality traits, such as anger and hostility^53^.

In marked contrast with the known behavioral consequences of *MAO-A* deficiency, the sequelae derived from absent or low *MAO-B* need further investigation in humans. In this regard, individuals suffering from Norrie disease (ND) have provided some valuable insights. ND is an inherited, X-linked eye disorder caused by a mutation in the *NDP* gene, which causes an atypical development of the retina resulting in blindness in male infants around the perinatal period^54^. These patients may also experience hearing and motor impairments, cognitive disability, along with other problems in basic physiological functions, such as breathing, digestion, or reproduction. ND patients harboring deletions or mutations affecting not only *NDP* but also *MAO-A* and/or *MAO-B* (which are neighboring genes to *NDP*) exhibit differential phenotypes; unlike patients with either *MAO-A* or combined *MAO-A/MAO-B* deficiency, patients with selective *MAO-B* deficiency (i.e., total lack of platelet MAO-B activity) failed to exhibit overt pathological behavior^55^. This result reinforces the specific role of *MAO-A* vs. *MAO-B* in the manifestations of clinical behavioral phenotypes and ultimately underscores the importance of syndromic cases to stimulate novel hypotheses and research in the biomedical field.

#### Neurobiological and anatomical correlates

Neuroimaging studies have explored the neurobiological underpinnings mediating aggression in carriers of low- vs. high-expressing alleles of the *MAO-A* gene. The low-expression variant, linked to increased risk of aggressive behavior, has been associated with structural and functional alterations in corticolimbic brain networks supporting emotional regulation and inhibitory control, in the prefrontal cortex, amygdala, and hippocampus^56,57^. In this line, a recent study found that healthy males carrying low-expressing alleles of the *MAO-A* gene exhibited differences in patterns of functional connectivity between brain areas responsible for emotional regulation, i.e., the dorsomedial prefrontal cortex, DMPFC, and empathy, i.e., the angular gyrus, AG), as compared with participants carrying high-expressing alleles of the gene^58^. Importantly, these neurobiological differences also mediated the relation of allele status and trait aggression in low-expressing allele carriers^58^. From a theoretical standpoint, Buckhold et al. suggested that increased aggression and aggressive traits in low-expressing allele carriers of the *MAO-A* gene might have a neurodevelopmental origin caused by the impact of excess serotonin in brain structures critical for social evaluation and emotional regulation^15^.

In *MAO-A*-deficient mice, morphological and functional abnormalities are found in different cortical and subcortical areas, including the PFC, amygdala, corpus callosum, and somatosensory cortex^13^. Interestingly, the increased aggression associated with *MAO-A* deficiency can be rescued by forebrain-specific, i.e., cortical expression of human MAO-A. This finding suggests that, as in humans, MAO-A levels in frontal cortical networks may underlie the expression of aggressive behaviors in *MAO-A* KO mice^59^.

### Outstanding questions and new perspectives

#### Gene–environment interactions, epigenetic factors, and demographic characteristics

As discussed above, experimental evidence in humans and rodents suggests that the promoter region of the *MAO-A* gene modulates behavioral manifestations of aggression. In humans, the core promoter region of *MAO-A* has been located in the two 90 bp repeat sequences, in turn comprised of four tandem repeats with a Sp1-binding site each. Indeed, the Sp1 family of proteins is one of the main factors controlling MAO-A expression in humans^23^. As discussed above, these studies highlight the mediating role of early trauma and other environmental stressors in this relation. In this regard, it remains unclear how the VNTR per se possibly interacts with other nearby DNA variants or other environmental factors (e.g., stress) to elicit aggressive responses or maladaptive personality traits since childhood^6,60^. Equally important would be to elucidate the potential effect of parental raising on MAO regulation and its protective effect in the face of early stressors^47^. Epigenetic factors, such as CpG methylation, seem to affect *MAO-A* mRNA expression at least in females, which in turn depend on *MAO-A* promoter polymorphisms^61^. Also, epigenetic alterations due to maternal care have been reported in the glucose transporter gene, genes affecting glutamate receptor activity, and *c-FOS*^62^, but not for the *MAO-A* or *MAO-B* genes. However, *c-FOS* has been speculatively associated with the prolonged expression of the *MAO* gene in chronic stress^63^. Given the link between MAO, stress reactivity, and aggression^63^, further research in animal models should address the effect of *MAO-A*-deficient parents in stress responses and modulation of aggressive behaviors in their offspring. Interestingly, evidence suggests that the epigenetic factors that modulate the interaction between *MAO-A* and aggression may be race-specific^64^; for instance, the aforementioned interaction between 2R- and 3R-repeat MAO-A variants (which, of note, are in contrast to 3.5R and 4R variants associated with higher transcriptional activity) and child abuse was associated with violence and antisocial behavior only in Caucasians but not in individuals from other ethnic group; however, as this study did not control for gender, it is likely its results could be pertinent to non-Caucasians, as well^65^; indeed, both genetic and environmental factors may underlie these racial differences^64^.

#### Physiological mechanisms

Though the relation between genetic variations in *MAO-A* and aggressive behavior has been addressed in several studies, the molecular and neural substrates underlying this association still remain obscure. A recent study suggested that *MAO-A* genotype determines brain and heart responses to aggression-inducing stimuli. Specifically, the authors found that healthy men carrying the 4.5-repeat allele variation, in comparison to those with 2.5- and 3.5-repeat variation, exhibited increased heart rate responses and a distinct neural oscillatory profile in response to visual scenes displaying aggressive behavior^66^. One possible interpretation of this finding is that altered serotoninergic metabolism caused by this allele variation may predispose individuals to a biased fight-or-flight response. In this scenario, ambiguous signals, such as increased heart rate may be more easily interpreted by the brain as threatening and hence trigger aggressive behaviors more easily. Similar investigations should focus on identifying new clinical phenotypes across *MAO-A* genotypes regarding violent behaviors and aggressive personality. It would be crucial to determine whether allelic variations in the *MAO-A* gene have a comparable impact on enzymatic activity, neurotransmission levels, neural activity, and behavior^66^.

#### Neurodevelopmental disorders

*MAO-A* deficiency has not only been exclusively linked to aggressive phenotypes but also to the developmental origin of sensory and communication deficits^13,45^. This observation underscores the phenotypic variability associated with *MAO-A*. In mice, *MAO-A* deficiency leads to sensorimotor, social, and communication deficits, which follow alterations in spontaneous behavior in the early postnatal period^45^. These features mimic the symptoms and developmental trajectory of autistic spectrum disorder (ASD). Although more data are necessary before extrapolating these results to humans, *MAO-A* deficiency was recently documented in a boy diagnosed with ASD and self-damaging behavior^11^. Moreover, boys diagnosed with ASD and having the low activity 3-repeat *MAOA* allele exhibited more severe symptomatology, including sensory impairment, arousal regulation problems, worse communication, and aggression, with aggressive behavior is also influenced by their mother’s genotype^67^. These results inspired the hypothesis that sensory and cognitive deficits in *MAO-A*-deficient children may aggravate aggressive and violent behaviors^13^. In addition, they justify the use of *MAO-A*-deficient animals as clinical models to understand these and other neurodevelopmental disorders characterized by aggressive phenotypes and lack of self-impulse control, including features of attention-deficit and hyperactivity disorder (ADHD)^68^.

#### MAO-A and the microbiota

In recent years, the role of the microbes in the gastrointestinal (GI) tract in brain physiology and behavior, including aggression, has gained substantial attention^69–71^. Importantly, gut bacteria can generate precursors of monoamine neurotransmitters directly involved in the modulation of aggression, including 5-HT^72^. In dogs, for instance, the gut microbiome was recently related to aggression, pointing to an aggression-related physiological state in interaction with the microbes in the GI tract^73^. In addition, we suggest that the influence that MAOIs exert on behavior may be in part mediated by the gut–brain axis, because MAOIs (a) react with the bacterial cofactors NAD and NADPH, and (b) have an antimicrobial effect by inhibiting cell wall synthesis^70,74^. In this line, studies in rodents have already shown that perturbations of microbiota as a result of antibiotic administration cause decreased aggressive behavior^75^. Finally, we know that the human microbiome may produce neuroactive compounds and, thus, affect behavior^76^. As is the case in the zebrafish^77^, human gut microbes may contain *mao* genes, which could alter the metabolism of serotonin and other amines in both the central nervous system and in the gut–brain axis. Although the role of the gut–brain axis in the MAO-related modulation of aggression and antisocial behavior is far from being understood, the aforementioned findings justify efforts in this direction.

#### Methodological considerations

The simultaneous measurement of multiple amine metabolizing molecules and the parallel combination of genetic, imaging, and issue-specific psychometric techniques, hold promise for the attainment of more definitive conclusions regarding the association between aggression and *MAO* deficiency per se, and/or in combination with other parameters, such as pharmacological sensitization, stressful environments, and concomitant genetic variations. In addition, the progression from self-reporting questionnaires to open phenotype characteristics assessed by neuropsychological tests will be an improvement in understanding personality genetics^78^. Ultimately, we propose a psycho–radio–genomic approach, where this question is resolved by combining multiple technologies from psychology, imaging, and genomics/genetics.

Regarding the specific role of the VNTR polymorphisms, it is crucial to determine the extent to which allelic variations in *MAO* genes are associated with enzymatic activity, and whether the latter equally influences antisocial or aggressive behavior in humans. For instance, the reported lack of association between *MAO-A* gene expression and brain MAO-A activity in healthy men suggests that the *MAO-A* gene promoter polymorphism does not contribute to differences in MAO-A activity^79^. Similarly, a *MAO-B* intron 13 polymorphism did not affect MAO-B activity in platelets^80^. These inconsistencies may be due to a lack of genotype-phenotype similarities among *MAO* gene alterations, an issue that should be taken under consideration in future studies.

Finally, future research should specifically address sex differences in the modulation of aggression by the MAO system, given the strong interactions between *MAO-A* polymorphisms and known key modulators of anger and aggression, most notably testosterone^81^.

## Translational and clinical implications

In this paper, we have summarized key findings linking MAOs and aggressive behavior in humans and animal models. To move the field forward, we have proposed that future investigations should address key outstanding questions about this connection, including (a) epigenetic factors modulating the impact of *MAO* genotype and VNTR on aggressive behaviors; (b) the effect of allelic variations in the *MAO-A* gene on enzymatic activity, neurotransmission, neural activity, and behavior; (c) the physiological evidence supporting MAO’s influence on aggressive behavior; (d) the effect of MAO-A deficiency on aggressive phenotypes in neurodevelopmental disorders characterized by sensory and communication deficits, including ASD; and, (e) the role of monoamines and the effects of MAOIs in different behaviors influenced by the microbiota in the bidirectional brain–gut axis. We anticipate that resolving these questions will enrich our understanding of the wide variety of neuropsychiatric disorders characterized by defects in the monoamine system^82^. Two interesting possibilities include discovering novel biomarkers of aggressive and violent behavior based on MAO activity, and targeting the MAO system to treat pathological aggression.

### Novel biomarkers of aggressive and violent behavior

Regarding *MAO-A*-related variations, saturation genome editing (SGE) can be implemented to provide further experimental evidence of the functional effects of *MAO-A* or other gene variants^83,84^. In SGE experiments, all possible single nucleotide variants are assayed in single targeted exons, thus, allowing functional classifications over a broad clinical spectrum^84^. As for gene products, analysis of monoamine neurotransmitters in CSF is an increasingly used practice in patients with motor deficits and may lead identification of disorders in which monoamine abnormalities are causative or part of the associated symptomatology^82^. This practice could be extended to patients exhibiting pathological aggression and antisocial behavior.

Schizophrenia and AD also offer an interesting framework to explore novel biomarkers and the role of MAO/MAOIs in agitation, irritability, and aggressive demeanor. For instance, it has been recently found that MAO-B platelet activity, but not *MAO-A* VNTR or *MAO-B* polymorphisms, was related to severe agitation in a sample of patients with schizophrenia and conduct disorder^85^. In AD, aggressive behavior is thought to be linked to serotoninergic impairment^86^. In this line, it would be crucial to determine if MAO platelet activity is associated with irritability and aggression in these patients, as was the case for self-rated verbal aggression in female patients with fibromyalgia^87^.

Finally, we would like to highlight the potential of imaging and tracing techniques to quantitatively map MAO brain activity in different clinical disorders characterized by aggressive behaviors and traits. For instance, PET/SPECT studies have assessed the expression of MAO-B, whose location is found in astrocytes’ external mitochondrial membrane, by using irreversible MAOIs in clinical populations^88^. Specifically, regarding pathological aggression, a PET study in antisocial personality disorder (ASPD) revealed decreased MAO-A levels compared to controls in different areas involved in impulse control and aggression, including the orbitofrontal cortex (OFC) and ventral striatum (VS)^89^. Given the promising value of such techniques, they should be applied to map abnormalities in the MAO system in subjects exhibiting different types of aggression and aggressive traits.

### Targeting the MAO system to treat pathological aggression

Pharmacological therapy has been widely used to control aggressive behaviors in a variety of clinical populations. Because of the role of MAOs and *MAO* genes in the manifestation of aggressive and antisocial behavior, drugs that target this system (e.g., MAOIs) have been used to this end. However, MAOIs have a global effect on behaviors that are unrelated to aggression, and this may cause unwanted side effects. In addition, a recent meta-analysis examining the efficacy of different interventions for agitation and aggression in patients with dementia showed that non-pharmacological interventions, i.e., behavioral therapy, multidisciplinary care, etc., were more efficacious in reducing aggression and agitation in adults than pharmacological therapy^90^.

Recent advances in the field offer promising avenues to target the MAO system without using pharmacological agents. For instance, Labonte and colleagues recently identified and described MAALIN, a novel long noncoding RNA (lncRNA), that regulates the activity of the MAO-A gene in the human brain^91^. In impulsive–aggressive individuals who committed suicide, epigenetic mechanisms regulating MAALIN expression in different brain areas were associated with MAOA expression. Taking this finding further, the authors demonstrated in mice that (a) driving MAALIN overexpression in neuroprogenitor cells decreased MAOA levels, while knocking out its expression led to elevated MAOA, and (b) hippocampal MAALIN decreased expression of MAOA and aggravated impulsive–aggressive behavior^91^. Although future research is essential to identify the exact epigenetic mechanisms involved in the regulation of MAALIN and other lncRNAs in clinical practice, these findings could lay the foundation for highly specific therapies for pathological aggression.

In animal models, cutting-edge techniques like opto- and chemogenetics hold the potential to identify new targets for the monoaminergic control of aggressive behavior. In so doing, gene targeting, gene overexpression, and chemo-/optogenetic modulation of monoaminergic brain centers could be applied to target the MAO system in selected pathways and assess its role in behavior^92,93^. For instance, a recent study identified a novel circuit comprised by the CA2 area of the hippocampus, the lateral septum, and the ventromedial hypothalamus, that modulates social aggression in mice^94^, whereas optogenetic activation of this circuit led to attacks, by silencing CA2 or CA2-LS projections that inhibit social aggression. In this context, it would be interesting to elucidate the specific role of MAO in this circuit in animal models of pathological aggression.

### The interplay between MAO and alterations in other neurobiological systems

When considering novel MAO-related biomarkers and therapeutic targets for aggression, we should take into account the influence of other systems that are tightly related to MAO and can have an impact on behavior. Of special interest is the interaction between MAO and glucocorticoids, which may be important in chronic stress states. In humans, *MAO-A* is targeted by glucocorticoids in both skeletal muscle and brain cells^95–97^, and activation of both the HPA axis and MAO-A is well-known in the acute stress response^96,98^. The bidirectional interaction between MAO and the HPA axis might be mediated by the effects of serotonin-related stimulation of the latter^99^. Indeed, acute stressors and administration of glucocorticoids decreased functional markers of MAO-A activity, including binding of monoamines, such as 5-HT to the active sites of the MAO-A enzyme, enzymatic activity, and enzyme protein levels^96^. Dysregulation of this physiological response or individual traits related to enhanced stress reactivity^100^ could give rise to maladaptive coping mechanisms, including aggression and violence, a hypothesis that warrants additional research.

## Concluding remarks

**Fig. 1:**
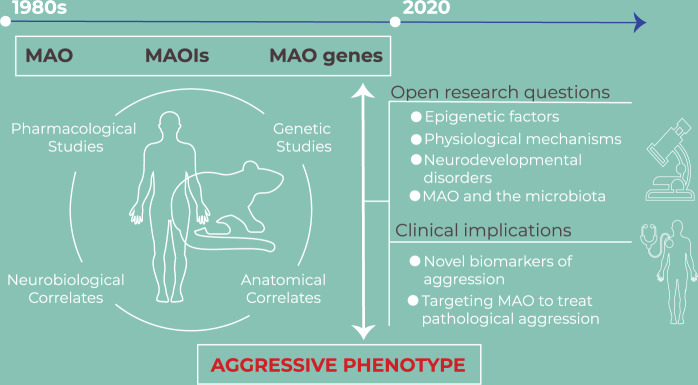
Graphical abstract on the associations between Monoamine Oxidases and aggressive behaviour. MAO: monoamine oxidases; MAOIs: monoamine oxidases inhibitors.

The overarching goal of this review was to offer an updated commentary on the role of the MAO system in the modulation of aggression. Using earlier and recent discoveries as a foundation, including pharmacological, genetic, neurobiological, and anatomical studies, we propose novel research questions that remain unanswered in the field, as well as the potential translational solutions derived from these findings (Fig. 1). Specifically, future investigations should focus on the epigenetic factors and physiological mechanisms mediating the role of MAO in aggression, whether alterations in the MAO system underlie aggressive phenotypes in neurodevelopmental disorders and the specific brain–gut pathways that contribute to this phenomenon. Elucidating these mechanisms will undoubtedly open novel avenues for the detection of novel biomarkers of aggression and therapies focusing on targeting the MAO system to curb pathological aggression, including genome editing^99^, epigenomic^100^, and other precision medicine approaches^110^, especially for vulnerable age groups, such as adolescents.

## References

[1] Marquand, A. F. et al. Conceptualizing mental disorders as deviations from normative functioning. *Mol. Psychiatry*10.1038/s41380-019-0441-1 (2019). 10.1038/s41380-019-0441-1 PMC6756106 31201374

[2] Olivier, B. & Young, L. Animal models of aggression. *Neuropsychopharmacol. Fifth Gener. Prog*. **118**, 1699–1708 (2002).

[3] Bandura, A. Social learning theory of aggression. *J. Commun*. 10.1111/j.1460-2466.1978.tb01621.x (1978). 10.1111/j.1460-2466.1978.tb01621.x 690254

[4] Bond, M. H. Culture and aggression—from context to coercion. *Personal. Soc. Psychol. Rev*. 10.1207/s15327957pspr0801_3 (2004). 10.1207/s15327957pspr0801_3 15121540

[5] Rosell, D. R. & Siever, L. J. The neurobiology of aggression and violence. *CNS Spectr*. 10.1017/S109285291500019X (2015). 10.1017/S109285291500019X 25936249

[6] Waltes, R., Chiocchetti, A. G. & Freitag, C. M. The neurobiological basis of human aggression: a review on genetic and epigenetic mechanisms. *Am. J. Med. Genet. Part B Neuropsychiatr. Genet*. 10.1002/ajmg.b.32388 (2016). 10.1002/ajmg.b.32388 26494515

[7] Anholt, R. R. H. & Mackay, T. F. C. Genetics of aggression. *Annu Rev. Genet.*10.1146/annurev-genet-110711-155514 (2012). 10.1146/annurev-genet-110711-155514 22934647

[8] Veroude, K. et al. Genetics of aggressive behavior: an overview. *Am. J. Med Genet Part B Neuropsychiatr. Genet*. 10.1002/ajmg.b.32364 (2016). 10.1002/ajmg.b.32364 26345359

[9] Freudenberg, F., Carreño Gutierrez, H., Post, A. M., Reif, A. & Norton, W. H. Aggression in non-human vertebrates: genetic mechanisms and molecular pathways. *Am. J. Med. Genet. Part B Neuropsychiatr. Genet*. 10.1002/ajmg.b.32358 (2016). 10.1002/ajmg.b.32358 26284957

[10] Brunner, H. G., Nelen, M., Breakefield, X. O., Ropers, H. H. & Van Oost, B. A. Abnormal behavior associated with a point mutation in the structural gene for monoamine oxidase A. *Science*10.1126/science.8211186 (1993). 10.1126/science.8211186 8211186

[11] Piton, A. et al. 20 ans après: a second mutation in MAOA identified by targeted high-throughput sequencing in a family with altered behavior and cognition. *Eur. J. Hum. Genet*. 10.1038/ejhg.2013.243 (2014). 10.1038/ejhg.2013.243 PMC4023218 24169519

[12] Palmer, E. E. et al. New insights into Brunner syndrome and potential for targeted therapy. *Clin. Genet*. 10.1111/cge.12589 (2016). 10.1111/cge.12589 25807999

[13] Godar, S. C., Fite, P. J., McFarlin, K. M. & Bortolato, M. The role of monoamine oxidase A in aggression: current translational developments and future challenges. *Prog. Neuro-Psychopharmacol. Biol. Psychiatry*10.1016/j.pnpbp.2016.01.001 (2016). 10.1016/j.pnpbp.2016.01.001 PMC4865459 26776902

[14] Popova, N. K. From genes to aggressive behavior: the role of serotonergic system. *BioEssays*10.1002/bies.20412 (2006). 10.1002/bies.20412 16615082

[15] Buckholtz, J. W. & Meyer-Lindenberg, A. MAOA and the neurogenetic architecture of human aggression. *Trends Neurosci*. 10.1016/j.tins.2007.12.006 (2008). 10.1016/j.tins.2007.12.006 18258310

[16] Vassos, E., Collier, D. A. & Fazel, S. Systematic meta-analyses and field synopsis of genetic association studies of violence and aggression. *Mol. Psychiatry*10.1038/mp.2013.31 (2014). 10.1038/mp.2013.31 PMC3965568 23546171

[17] Ficks, C. A. & Waldman, I. D. Candidate genes for aggression and antisocial behavior: a meta-analysis of association studies of the 5HTTLPR and MAOA-uVNTR. *Behav. Genet*. 10.1007/s10519-014-9661-y (2014). 10.1007/s10519-014-9661-y 24902785

[18] Ruisch, I. H., Dietrich, A., Glennon, J. C., Buitelaar, J. K. & Hoekstra, P. J. Interplay between genome-wide implicated genetic variants and environmental factors related to childhood antisocial behavior in the UK ALSPAC cohort. *Eur. Arch. Psychiatry Clin. Neurosci*. 10.1007/s00406-018-0964-5 (2019). 10.1007/s00406-018-0964-5 PMC6689282 30569215

[19] Scott, A. L., Bortolato, M., Chen, K. & Shih, J. C. Novel monoamine oxidase A knock out mice with human-like spontaneous mutation. *Neuroreport*10.1097/WNR.0b013e3282fd6e88 (2008). 10.1097/WNR.0b013e3282fd6e88 PMC3435113 18418249

[20] Sohrabi, S. The criminal gene: the link between MAOA and aggression (REVIEW). *BMC Proc*. 10.1186/1753-6561-9-s1-a49 (2015).

[21] Takahashi, A., Quadros, I. M., de Almeida, R. M. M. & Miczek, K. A. Behavioral and pharmacogenetics of aggressive behavior. *Curr. Top. Behav. Neurosci*. 10.1007/7854_2011_191 (2012). 10.1007/7854_2011_191 PMC3864145 22297576

[22] Fierro, A. et al. Similarities between the binding sites of monoamine oxidase (MAO) from different species — is zebrafish a useful model for the discovery of novel MAO inhibitors? In *An Integrated View of the Molecular Recognition and Toxinology—From Analytical Procedures to Biomedical Applications.* (ed. Radis-Baptista, G.) 405–431 (InTech—Open Access Publisher, 2013).

[23] Zhu, Q. S., Chen, K. & Shih, J. C. Bidirectional promoter of human monoamine oxidase A (MAO A) controlled by transcription factor Sp1. *J. Neurosci*. 10.1523/jneurosci.14-12-07393.1994 (1994). 10.1523/JNEUROSCI.14-12-07393.1994 PMC6576875 7996184

[24] Wong, W. K., Ou, X. M., Chen, K. & Shih, J. C. Activation of human monoamine oxidase B gene expression by a protein kinase C MAPK signal transduction pathway involves c-Jun and Egr-1. *J. Biol. Chem*. 10.1074/jbc.M202844200 (2002). 10.1074/jbc.M202844200 PMC2861899 11956220

[25] Shih, J. C. Monoamine oxidase isoenzymes: genes, functions and targets for behavior and cancer therapy. *J. Neural Transm*. 10.1007/s00702-018-1927-8 (2018). 10.1007/s00702-018-1927-8 PMC6245662 30259128

[26] Youdim, M. B. H., Edmondson, D. & Tipton, K. F. The therapeutic potential of monoamine oxidase inhibitors. *Nat. Rev. Neurosci*. 10.1038/nrn1883 (2006). 10.1038/nrn1883 16552415

[27] Edmondson, D. E. & Binda, C. Monoamine oxidases. *Subcell. Biochem*. 10.1007/978-981-10-7757-9_5 (2018). 10.1007/978-981-10-7757-9_5 29464559

[28] O’Carroll, A.-M., Fowler, C. J., Phillips, J. P., Tobbia, I. & Tipton, K. F. The deamination of dopamine by human brain monoamine oxidase. *Naunyn Schmiedebergs Arch. Pharm*. 10.1007/bf00500765 (1983). 10.1007/BF00500765 6408492

[29] Westlund, K. N., Denney, R. M., Kochersperger, L. M., Rose, R. M. & Abell, C. W. Distinct monoamine oxidase A and B populations in primate brain. *Science*10.1126/science.3875898 (1985). 10.1126/science.3875898 3875898

[30] Garrick, N. A. & Murphy, D. L. Species differences in the deamination of dopamine and other substrates for monoamine oxidase in brain. *Psychopharmacology*10.1007/BF00433804 (1980). 10.1007/BF00433804 6781004

[31] Katsouni, E., Sakkas, P., Zarros, A., Skandali, N. & Liapi, C. The involvement of substance P in the induction of aggressive behavior. *Peptides*10.1016/j.peptides.2009.05.001 (2009). 10.1016/j.peptides.2009.05.001 19442694

[32] Siever, L. J. Neurobiology of aggression and violence. *Am. J. Psychiatry*10.1176/appi.ajp.2008.07111774 (2008). 10.1176/appi.ajp.2008.07111774 PMC4176893 18346997

[33] Raj, Y. P. Psychopharmacology of borderline personality disorder. *Curr. Psychiatry Rep*. 10.1007/s11920-004-0068-y (2004). 10.1007/s11920-004-0068-y 15142476

[34] Kolla, N. J. & Vinette, S. A. Monoamine oxidase A in antisocial personality disorder and borderline personality disorder. *Curr. Behav. Neurosci. Rep*. 10.1007/s40473-017-0102-0 (2017). 10.1007/s40473-017-0102-0 PMC5846806 29568721

[35] Fowler, J. S. et al. Evidence that formulations of the selective MAO-B inhibitor, selegiline, which bypass first-pass metabolism, also inhibit MAO-A in the human brain. *Neuropsychopharmacology*10.1038/npp.2014.214 (2015). 10.1038/npp.2014.214 PMC4289953 25249059

[36] Mejia, J. M., Ervin, F. R., Baker, G. B. & Palmour, R. M. Monoamine oxidase inhibition during brain development induces pathological aggressive behavior in mice. *Biol. Psychiatry*10.1016/S0006-3223(02)01418-X (2002). 10.1016/s0006-3223(02)01418-x 12372653

[37] Yu, Q. et al. Dopamine and serotonin signaling during two sensitive developmental periods differentially impact adult aggressive and affective behaviors in mice. *Mol. Psychiatry*10.1038/mp.2014.10 (2014). 10.1038/mp.2014.10 PMC4311886 24589889

[38] Griebel, G., Curet, O., Perrault, G. & Sanger, D. J. Behavioral effects of phenelzine in an experimental model for screening anxiolytic and anti-panic drugs: correlation with changes in monoamine-oxidase activity and monoamine levels. *Neuropharmacology*10.1016/S0028-3908(98)00077-X (1998). 10.1016/s0028-3908(98)00077-x 9776388

[39] Duane Sofia, R. Structural relationship and potency of agents which selectively block mouse killing (muricide) behavior in rats. *Life Sci*. 10.1016/0024-3205(69)90049-6 (1969). 10.1016/0024-3205(69)90049-6 4391120

[40] Ossowska, G., Klenk-Majewska, B., Danilczuk, Z., Wróbel, A. & Zebrowska-Łupina, I. Reversal of stress-induced deficit in aggression by monoamine oxidase inhibitors. *Pol. J. Pharmacol.* **51**, 391–397 (1999). 10817539

[41] Kopin. I. J. The relationship of early studies of monoamine oxidase to present concepts. *J. Neural Transm. Suppl*. 10.1007/978-3-211-33328-0_9 (2006). 10.1007/978-3-211-33328-0_9 17447418

[42] Cases, O. et al. Aggressive behavior and altered amounts of brain serotonin and norepinephrine in mice lacking MAOA. *Science* (80-) 10.1126/science.7792602 (1995). 10.1126/science.7792602 PMC2844866 7792602

[43] Shih, J. C. & Chen, K. MAO-A and -B gene knock-out mice exhibit distinctly different behavior. *Neurobiology* **7**, 235–246 (1999). 10591056

[44] Bortolato. M. et al. Social deficits and perseverative behaviors, but not overt aggression, in MAO-A hypomorphic mice. *Neuropsychopharmacology*10.1038/npp.2011.157 (2011). 10.1038/npp.2011.157 PMC3230491 21832987

[45] Bortolato, M., Floris, G. & Shih, J. C. From aggression to autism: new perspectives on the behavioral sequelae of monoamine oxidase deficiency. *J. Neural Transm*. 10.1007/s00702-018-1888-y (2018). 10.1007/s00702-018-1888-y PMC6215718 29748850

[46] Sabol, S. Z., Hu, S. & Hamer, D. A functional polymorphism in the monoamine oxidase A gene promoter. *Hum. Genet*. 10.1007/s004390050816 (1998). 10.1007/s004390050816 9799080

[47] Caspi, A. et al. Role of genotype in the cycle of violence in maltreated children. *Science*10.1126/science.1072290 (2002). 10.1126/science.1072290 12161658

[48] Fergusson, D. M., Boden, J. M., Horwood, L. J., Miller, A. L. & Kennedy, M. A. MAOA abuse exposure and antisocial behaviour: 30-Year longitudinal study. *Br. J. Psychiatry*10.1192/bjp.bp.110.086991 (2011). 10.1192/bjp.bp.110.086991 PMC3105117 21628708

[49] Frazzetto, G. et al. Early trauma and increased risk for physical aggression during adulthood: the moderating role of MAOA genotype. PLoS ONE 10.1371/journal.pone.0000486 (2007). 10.1371/journal.pone.0000486 PMC1872046 17534436

[50] Sjöberg, R. L. et al. Adolescent girls and criminal activity: Role of MAOA-LPR genotype and psychosocial factors. *Am. J. Med. Genet. Part B Neuropsychiatr. Genet*. 10.1002/ajmg.b.30360 (2007). 10.1002/ajmg.b.30360 17034017

[51] Sjöberg, R. L. et al. A non-additive interaction of a functional MAO-A VNTR and testosterone predicts antisocial behavior. *Neuropsychopharmacology*10.1038/sj.npp.1301417 (2008). 10.1038/sj.npp.1301417 PMC2665792 17429405

[52] Alia-Klein, N. et al. Brain monoamine oxidase A activity predicts trait aggression. *J. Neurosci*. 10.1523/JNEUROSCI.0925-08.2008 (2008). 10.1523/JNEUROSCI.0925-08.2008 PMC2430409 18463263

[53] Soliman, A. et al. Relationship of monoamine oxidase A binding to adaptive and maladaptive personality traits. *Psychol. Med*. 10.1017/S0033291710001601 (2011). 10.1017/S0033291710001601 20810002

[54] Berger, W. et al. Isolation of a candidate gene for Norrie disease by positional cloning. *Nat. Genet*. 10.1038/ng0692-199 (1992). 10.1038/ng0692-199 1303235

[55] Lenders, J. W. M. et al. Specific genetic deficiencies of the A and B isoenzymes of monoamine oxidase are characterized by distinct neurochemical and clinical phenotypes. *J. Clin. Investig*. 10.1172/JCI118492 (1996). 10.1172/JCI118492 PMC507147 8613523

[56] Meyer-Lindenberg, A. et al. Neural mechanisms of genetic risk for impulsivity and violence in humans. *Proc. Natl Acad. Sci. USA*10.1073/pnas.0511311103 (2006). 10.1073/pnas.0511311103 PMC1458867 16569698

[57] Harneit, A. et al. MAOA-VNTR genotype affects structural and functional connectivity in distributed brain networks. *Hum. Brain Mapp*. 10.1002/hbm.24766 10.1002/hbm.24766 PMC6864897 31441562

[58] Klasen, M. et al. Neural networks underlying trait aggression depend on MAOA gene alleles. *Brain Struct. Funct*. 10.1007/s00429-017-1528-6 (2018). 10.1007/s00429-017-1528-6 29019036

[59] Chen, K. et al. Forebrain-specific expression of monoamine oxidase A reduces neurotransmitter levels, restores the brain structure, and rescues aggressive behavior in monoamine oxidase A-deficient mice. *J. Biol. Chem*. 10.1074/jbc.M609830200 (2007). 10.1074/jbc.M609830200 PMC2844870 17090537

[60] Beitchman. J. H., Mik, H. M., Ehtesham, S., Douglas, L. & Kennedy, J. L. MAOA and persistent, pervasive childhood aggression. *Mol. Psychiatry*10.1038/sj.mp.4001492 (2004). 10.1038/sj.mp.4001492 15024395

[61] Pinsonneault, J. K., Papp, A. C. & Sadée, W. Allelic mRNA expression of X-linked monoamine oxidase a (MAOA) in human brain: Dissection of epigenetic and genetic factors. *Hum. Mol. Genet.*10.1093/hmg/ddl192 (2006). 10.1093/hmg/ddl192 16893905

[62] Fish, E. W. et al. Epigenetic programming of stress responses through variations in maternal care. *Ann. N. Y. Acad. Sci*. 10.1196/annals.1330.011 (2004). 10.1196/annals.1330.011 15817737

[63] Craig, I. W. The importance of stress and genetic variation in human aggression. *BioEssays*10.1002/bies.20538 (2007). 10.1002/bies.20538 17295220

[64] Kolla, N. J. & Bortolato, M. The role of monoamine oxidase A in the neurobiology of aggressive, antisocial, and violent behavior: a tale of mice and men*. Prog. Neurobiol.*10.1016/j.pneurobio.2020.101875 (2020). 10.1016/j.pneurobio.2020.101875 PMC7609507 32574581

[65] Widom. C. S. & Brzustowicz, L. M. MAOA and the ‘cycle of violence:’ childhood abuse and neglect, MAOA genotype, and risk for violent and antisocial behavior. *Biol. Psychiatry*10.1016/j.biopsych.2006.03.039 (2006). 10.1016/j.biopsych.2006.03.039 16814261

[66] Im, S. Y. et al. MAOA variants differ in oscillatory EEG & ECG activities in response to aggression-inducing stimuli. *Sci. Rep*. 10.1038/s41598-019-39103-7 (2019). 10.1038/s41598-019-39103-7 PMC6390082 30804379

[67] Cohen, I. L. et al. Autism severity is associated with child and maternal MAOA genotypes. *Clin. Genet.* **79**, 355–362 (2011). 10.1111/j.1399-0004.2010.01471.x 20573161

[68] Brookes, K. et al. The analysis of 51 genes in DSM-IV combined type attention deficit hyperactivity disorder: association signals in DRD4, DAT1 and 16 other genes. *Mol. Psychiatry*10.1038/sj.mp.4001869 (2006). 10.1038/sj.mp.4001869 16894395

[69] Sherwin, E., Bordenstein, S. R., Quinn, J. L., Dinan, T. G. & Cryan, J. F. Microbiota and the social brain. *Science*10.1126/science.aar2016 (2019). 10.1126/science.aar2016 31672864

[70] Evrensel, A., Önen Ünsalver, B. & Ceylan, M. E. Therapeutic potential of the microbiome in the treatment of neuropsychiatric disorders. *Med. Sci*. 10.3390/medsci7020021 (2019). 10.3390/medsci7020021 PMC6410187 30709065

[71] Hulme, H. et al. Microbiome-derived carnitine mimics as previously unknown mediators of gut-brain axis communication. *Sci. Adv*. 10.1126/sciadv.aax6328 (2020). 10.1126/sciadv.aax6328 PMC7065903 32195337

[72] O’Mahony, S. M., Clarke, G., Borre, Y. E., Dinan, T. G. & Cryan, J. F. Serotonin, tryptophan metabolism and the brain-gut-microbiome axis. *Behav. Brain Res*. 10.1016/j.bbr.2014.07.027 (2015). 10.1016/j.bbr.2014.07.027 25078296

[73] Kirchoff, N. S., MAR, Udell & Sharpton, T. J. The gut microbiome correlates with conspecific aggression in a small population of rescued dogs (Canis familiaris). *PeerJ*10.7717/peerj.6103 (2019). 10.7717/peerj.6103 PMC6330041 30643689

[74] Macedo, D. et al. Antidepressants, antimicrobials or both? Gut microbiota dysbiosis in depression and possible implications of the antimicrobial effects of antidepressant drugs for antidepressant effectiveness. *J. Affect. Disord*. 10.1016/j.jad.2016.09.012 (2017). 10.1016/j.jad.2016.09.012 27744123

[75] Sylvia K. E, Rendon N. M, St John E. A, Demas G. E. Repeated antibiotic treatment affects social behaviors in Siberian Hamsters (*Phodopus sungorus*). FASEB J. 30(1_supplement), 1027–3 (2016).

[76] Dinan, T. G., Stilling, R. M., Stanton, C. & Cryan, J. F. Collective unconscious: how gut microbes shape human behavior. *J. Psychiatr. Res*. 10.1016/j.jpsychires.2015.02.021 (2015). 10.1016/j.jpsychires.2015.02.021 25772005

[77] Borrelli, L. et al. Probiotic modulation of the microbiota-gut-brain axis and behaviour in zebrafish. *Sci. Rep*. 10.1038/srep30046 (2016). 10.1038/srep30046 PMC4945902 27416816

[78] Ebstein, R. P. The molecular genetic architecture of human personality: beyond self-report questionnaires. *Mol. Psychiatry*10.1038/sj.mp.4001814 (2006). 10.1038/sj.mp.4001814 16534505

[79] Fowler, J. S. et al. Evidence that brain MAO A activity does not correspond to MAO A genotype in healthy male subjects. *Biol. Psychiatry*10.1016/j.biopsych.2006.08.038 (2007). 10.1016/j.biopsych.2006.08.038 PMC2712611 17141746

[80] Pivac, N. et al. The lack of association between monoamine oxidase (MAO) intron 13 polymorphism and platelet MAO-B activity among men. *Life Sci*. 10.1016/j.lfs.2005.12.030 (2006). 10.1016/j.lfs.2005.12.030 16427095

[81] Wagels, L. et al. Exogenous testosterone and the monoamine-oxidase A polymorphism influence anger, aggression and neural responses to provocation in males. *Neuropharmacology*10.1016/j.neuropharm.2019.01.006 (2019). 10.1016/j.neuropharm.2019.01.006 30639342

[82] Kurian, M. A., Gissen, P., Smith, M., Heales, S. J. R. & Clayton, P. T. The monoamine neurotransmitter disorders: an expanding range of neurological syndromes. *Lancet Neurol*. 10.1016/S1474-4422(11)70141-7 (2011). 10.1016/S1474-4422(11)70141-7 21777827

[83] Findlay, G. M., Boyle, E. A., Hause, R. J., Klein, J. C. & Shendure, J. Saturation editing of genomic regions by multiplex homology-directed repair. *Nature*10.1038/nature13695 (2014). 10.1038/nature13695 PMC4156553 25141179

[84] Findlay, G. M. et al. Accurate classification of BRCA1 variants with saturation genome editing. *Nature*10.1038/s41586-018-0461-z (2018). 10.1038/s41586-018-0461-z PMC6181777 30209399

[85] Nikolac Perkovic, M. et al. Monoamine oxidase and agitation in psychiatric patients. *Prog. Neuropsychopharmacol. Biol. Psychiatry*10.1016/j.pnpbp.2016.02.002 (2016). 10.1016/j.pnpbp.2016.02.002 26851573

[86] Zarros, A. C., Kalopita, K. S. & Tsakiris, S. T. Serotoninergic impairment and aggressive behavior in Alzheimer’s disease. *Acta Neurobiol. Exp.* **65**, 277–286 (2005). 10.55782/ane-2005-1563 16130802

[87] Prochazka, H., Anderberg, U. M., Oreland, L., Von Knorring, L. & Ågren, H. Self-rated aggression related to serum testosterone and platelet MAO activity in female patients with the fibromyalgia syndrome. *World J. Biol. Psychiatry*10.3109/15622970309167909 (2003). 10.3109/15622970309167909 12582976

[88] Valotassiou, V. et al. Clinical evaluation of brain perfusion SPECT with Brodmann areas mapping in early diagnosis of Alzheimer’s disease. *J. Alzheimer’s Dis*. 10.3233/JAD-150068 (2015). 10.3233/JAD-150068 26401711

[89] Kolla, N. J. et al. Lower monoamine oxidase-a total distribution volume in impulsive and violent male offenders with antisocial personality disorder and high psychopathic traits: an ^11^C Harmine positron emission tomography study. *Neuropsychopharmacology*10.1038/npp.2015.106 (2015). 10.1038/npp.2015.106 PMC4569949 26081301

[90] Watt, J. A. et al. Comparative efficacy of interventions for aggressive and agitated behaviors in dementia. *Ann. Intern Med*. 10.7326/m19-0993 (2019). 10.7326/M19-0993 31610547

[91] Labonté, B. et al. Regulation of impulsive and aggressive behaviours by a novel lncRNA. *Mol. Psychiatry*10.1038/s41380-019-0637-4 (2020). 10.1038/s41380-019-0637-4 PMC7436429 31907380

[92] Lőrincz, M. L. & Adamantidis, A. R. Monoaminergic control of brain states and sensory processing: existing knowledge and recent insights obtained with optogenetics. *Prog. Neurobiol*. 10.1016/j.pneurobio.2016.09.003 (2017). 10.1016/j.pneurobio.2016.09.003 27634227

[93] Runegaard, A. H. et al. Modulating dopamine signaling and behavior with chemogenetics: concepts, progress, and challenges. *Pharm. Rev*. 10.1124/pr.117.013995 (2019). 10.1124/pr.117.013995 30814274

[94] Leroy, F. et al. A circuit from hippocampal CA2 to lateral septum disinhibits social aggression. *Nature*10.1038/s41586-018-0772-0 (2018). 10.1038/s41586-018-0772-0 PMC6364572 30518859

[95] Manoli, I. et al. Monoamine oxidase-A is a major target gene for glucocorticoids in human skeletal muscle cells. *FASEB J*. 10.1096/fj.04-3660fje (2005). 10.1096/fj.04-3660fje 15946989

[96] Soliman, A. et al. Convergent effects of acute stress and glucocorticoid exposure upon MAO-A in humans. *J. Neurosci*. 10.1523/JNEUROSCI.2091-12.2012 (2012). 10.1523/JNEUROSCI.2091-12.2012 PMC6621843 23197705

[97] Manoli, I. et al. Mitochondria as key components of the stress response. *Trends Endocrinol. Metab*. 10.1016/j.tem.2007.04.004 (2007). 10.1016/j.tem.2007.04.004 17500006

[98] Higuchi, Y., Soga, T. & Parhar, I. S. Regulatory pathways of monoamine oxidase A during social stress. *Front. Neurosci*. 10.3389/fnins.2017.00604 (2017). 10.3389/fnins.2017.00604 PMC5671571 29163009

[99] Calogero, A. E. et al. Mechanisms of serotonin receptor agonist-induced activation of the hypothalamic-pituitary-adrenal axis in the rat. *Endocrinology*10.1210/endo-126-4-1888 (1990). 10.1210/endo-126-4-1888 2156670

[100] Singh, A., Petrides, J. S., Gold, P. W., Chrousos, G. P. & Deuster, P. A. Differential hypothalamic-pituitary-adrenal axis reactivity to psychological and physical stress. *J. Clin. Endocrinol. Metab*. 10.1210/jc.84.6.1944 (1999). 10.1210/jcem.84.6.5746 10372691

[101] Ramirez, J. M. Animal models in the research of human aggression. *Aggress. Violent Behav*. 10.1016/S1359-1789(99)00009-9 (2000).

[102] Southwick, C. H. In *The Psychobiology of Aggression.* (ed. Moyer, K. E.) xviii + 402 (Harper and Row, 1976).

[103] Takahashi, A. & Miczek, K. A. Neurogenetics of aggressive behavior: studies in rodents. *Curr. Top. Behav. Neurosci*. 10.1007/7854_2013_263 (2015). 10.1007/7854_2013_263 PMC4092042 24318936

[104] Krahé, B. *The Social Psychology of Aggression*, second edn. (Routledge, 2013).

[105] Bushman, B. J. & Anderson, C. A. Is it time to pull the plug on the hostile versus instrumental aggression dichotomy? *Psychol. Rev*. 10.1037/0033-295X.108.1.273 (2001). 10.1037/0033-295x.108.1.273 11212630

[106] Wranghama, R. W. Two types of aggression in human evolution. *Proc. Natl Acad. Sci. USA*10.1073/pnas.1713611115 (2017). 10.1073/pnas.1713611115 PMC5777045 29279379

[107] Siegel, A. E. & Buss, A. H. The psychology of aggression. *Am. J. Psychol*. 10.2307/1419735 (1963).

[108] Waltes, R., Chiocchetti, A. G. & Freitag, C. M. The neurobiological basis of human aggression: a review on genetic and epigenetic mechanisms. *Am. J. Med. Genet. Part B Neuropsychiatr. Genet*. 10.1002/ajmg.b.32388 (2016). 10.1002/ajmg.b.32388 26494515

[109] Gary R. Vandenbos. APA Dictionary of Psychology, 2nd edn. 10.1037/14646-000 (The American Psychological Association, 2016).

[110] Mentis A. A, Pantelidi K, Dardiotis E, Hadjigeorgiou G. M, Petinaki E. Precision Medicine and Global Health: The Good, the Bad, and the Ugly. Front Med (Lausanne) **14**, 67, 10.3389/fmed.2018.00067 (2018). 10.3389/fmed.2018.00067 PMC5861134 29594124

