# Correction: From *warrior genes* to translational solutions: novel insights into monoamine oxidases (MAOs) and aggression

**DOI:** 10.1038/s41398-021-01611-4

**Published:** 2021-09-22

**Authors:** Alexios-Fotios A. Mentis, Efthimios Dardiotis, Eleni Katsouni, George P. Chrousos

**Affiliations:** 1 Public Health Laboratories, Hellenic Pasteur Institute, Vas. Sofias Avenue 127, 115 21 Athens, Greece; 2 Department of Neurology, University of Thessaly, Panepistimiou 3, Viopolis, 41 500 Larissa, Greece; 3 Department of Experimental Psychology, Oxford University, Oxford, UK; 4 University Research Institute of Maternal and Child Health and Precision Medicine, National and Kapodistrian University of Athens, Medical School, Aghia Sophia Children’s Hospital, Livadias 8, 115 27 Athens, Greece; 5 UNESCO Chair on Adolescent Health Care, Athens, Greece

**Keywords:** Molecular neuroscience, Psychiatric disorders, Clinical pharmacology

Correction to: *Translational Psychiatry* 10.1038/s41398-021-01257-2, published online 18 February 2021

The original version of this article unfortunately contained an error. The authors recommend adding a phrase at the end of the section “Gene–environment interactions, epigenetic factors, and demographic characteristics”.

In doing so, the phrase “for instance, the aforementioned interaction between 2R- and 3R-repeat MAO-A variants (which, of note, are in contrast to 3.5R and 4R variants associated with higher transcriptional activity) and child abuse was associated with violence and antisocial behavior only in Caucasians but not in individuals from other ethnic group” changes to “for instance, the aforementioned interaction between 2R- and 3R-repeat MAO-A variants (which, of note, are in contrast to 3.5R and 4R variants associated with higher transcriptional activity) and child abuse was associated with violence and antisocial behavior only in Caucasians but not in individuals from other ethnic group; however, as this study did not control for gender, it is likely its results could be pertinent to non-Caucasians, as well”. The original article has been corrected.

